# A New Hope in Immunotherapy for Malignant Gliomas: Adoptive T Cell Transfer Therapy

**DOI:** 10.1155/2014/326545

**Published:** 2014-06-09

**Authors:** Dong-Sup Chung, Hye-Jin Shin, Yong-Kil Hong

**Affiliations:** ^1^Department of Neurosurgery, Incheon St. Mary's Hospital, The Catholic University of Korea College of Medicine, Dongsuro 56, Bupyeong-gu, Incheon 403-720, Republic of Korea; ^2^Department of Neurosurgery, Seoul St. Mary's Hospital, The Catholic University of Korea College of Medicine, Banpodaero 222, Seocho-gu, Seoul 137-701, Republic of Korea

## Abstract

Immunotherapy emerged as a promising therapeutic approach to highly incurable malignant gliomas due to tumor-specific cytotoxicity, minimal side effect, and a durable antitumor effect by memory T cells. But, antitumor activities of endogenously activated T cells induced by immunotherapy such as vaccination are not sufficient to control tumors because tumor-specific antigens may be self-antigens and tumors have immune evasion mechanisms to avoid immune surveillance system of host. Although recent clinical results from vaccine strategy for malignant gliomas are encouraging, these trials have some limitations, particularly their failure to expand tumor antigen-specific T cells reproducibly and effectively. An alternative strategy to overcome these limitations is adoptive T cell transfer therapy, in which tumor-specific T cells are expanded *ex vivo* rapidly and then transferred to patients. Moreover, enhanced biologic functions of T cells generated by genetic engineering and modified immunosuppressive microenvironment of host by homeostatic T cell expansion and/or elimination of immunosuppressive cells and molecules can induce more potent antitumor T cell responses and make this strategy hold promise in promoting a patient response for malignant glioma treatment. Here we will review the past and current progresses and discuss a new hope in adoptive T cell therapy for malignant gliomas.

## 1. Introduction

The prognosis of malignant glioma patients is grim despite the advanced multimodality therapies including surgery, radiotherapy, and chemotherapy. Immunotherapy emerged as a potential therapeutic approach to the highly incurable malignant gliomas, for which, however, either encouraging results or disappointing limitations were revealed as an alternative strategy [1, 2].

Tumor-specific CD8^+^cytotoxic T lymphocytes (CTLs) are generated by repetitive stimulation of peripheral blood mononuclear cells (PBMCs) with tumor-associated antigen (TAA) expressing antigen-presenting cells (APC) such as dendritic cells (DCs) and certain cytokines including interleukin- (IL-) 2, IL-7, IL-12, IL-15, and IL-21 [3, 4]. These cells can be expanded rapidly* ex vivo* to use them for adoptive cell therapy (ACT). Antigen sources for this procedure include major histocompatibility complex- (MHC-) restricted peptides, recombinant proteins, tumor lysates, and genetically introduced tumor antigen genes. CD4^+^ T cells may also exert antitumor effector functions mainly through the secretion of interferon- (IFN-) *γ* [5].

Theoretically, tumor-specific CTLs can move to TAA-overexpressed tumor cells specifically and kill them without adverse effects on normal cells. But, immune system may recognize these TAAs as self-antigens, leading to decreased T cell response to tumor cells because TAAs are also somewhat expressed in normal tissues [6, 7]. T cells with high affinity to self-antigen may be physiologically removed through the mechanisms of immune tolerance, so the endogenously activated tumor-specific T cells have low affinity to self-antigen, inducing limited T cell response [8]. Furthermore, tumors have evolved numerous mechanisms to evade both innate and adaptive immunity. These include modulation of MHC antigens and costimulatory molecules, expression of Fas ligand and other apoptotic molecules on the cell surface, production of inhibitory molecules such as transforming growth factor- (TGF-) *β* and IL-10, constitutive expression of the tryptophan-depleting enzyme, indoleamine 2,3-dioxygenase (IDO), and recruitment of regulatory T cells (Tregs) [9].

**Figure 1:**
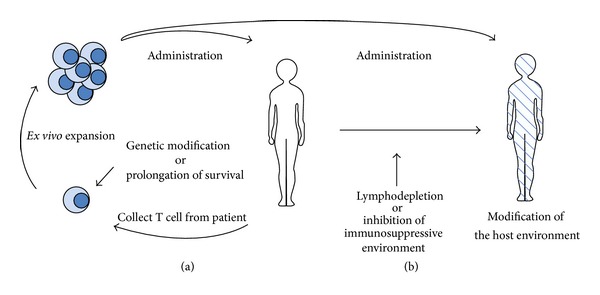
Adoptive T cell transfer therapy. (a) Enhancement of tumor-specific T cell function. (b) Modification of the host environment.

Results from recent immunotherapeutic clinical trials with tumor cell or DC vaccines for malignant glioma patients were encouraging [10–13]. These trials, however, have shown some limitations, particularly their failure to expand tumor antigen-specific T cells reproducibly and effectively, suggesting that endogenous activation of T cells is insufficient to control tumors. A strategy to overcome these limitations is adoptive T cell transfer, in which tumor-specific T cells are expanded* ex vivo* rapidly and then transferred to patients. Moreover, a recent advance in delivering therapeutic genes into somatic cells has been applicable to T cell therapy for tumors. T cells used in ACT can be modified to increase their specificity and survival for the tumor or to make them resistant to immune evasion mechanisms [14–25] (Figure 1). T cell response for malignant gliomas also can be improved by combination with other therapeutic modalities [26, 27].

Here we will review past experiences and discuss current promising strategies of adoptive T cell therapy for malignant gliomas.

## 2. Immune Environment of Malignant Glioma

The brain has long been considered to be immunologically privileged due to immediate inability to reject intracranial xenograft in early report [28], physical isolation from the systemic immune system by the blood-brain-barrier (BBB), and lack of connections to the lymphatic system. Subsequent studies, however, have described the efficient rejection of intracranial xenografts and allografts in immunocompetent hosts abundantly [29], capability of activated T cells to cross the BBB [30, 31], and the drainage of cerebrospinal fluid into systemic lymphatics [32]. In addition, no specific CNS-associated antigens have been known that are systematically immunogenic but evade immune surveillance within the brain unlike testes, other immunologically privileged site [33]. Microglia, resident APCs in the brain, play a crucial role in the CNS immune response [34]. Collectively, these results clearly indicate that the brain is not an immunologically privileged site, but may be an organ that has immunologically particular environment although not fully understood.

A critical step for an efficient stimulation of adaptive immune response even in the brain is the identification of suitable tumor-specific or tumor-associated antigens that can be recognized and eliminated by the immune system. Malignant glioma is known to be genetically heterogenous with a variety of antigen profile [48], so glioma cells are inefficient for antigen processing. Difficulty in identification of ideal tumor antigens for immunotherapy as well as the above-mentioned immune evasion mechanisms and the presence of immune inhibitory cells may render malignant glioma resistant to T cell responses. The source of antigen used in initial immunotherapeutic approaches to the malignant glioma was tumor lysates derived from autologous irradiated glioma cells [50]. Numerous glioma-associated antigens have been identified over the past decades and the antigens most suitable for activating the host-specific T cell response are still under investigation (Table 1). The glioma-specific antigens used in recent preclinical or clinical studies showing potent antiglioma effect include IL-13R*α*2, human epidermal growth factor receptor 2 (HER2), epidermal growth factor receptor variant III (EGFRvIII), and erythropoietin-producing hepatocellular carcinoma A2 (EphA2) [19–22, 51].

Recruitment of lymphocytes is a key of immune response. Immune cells can infiltrate to malignant glioma at later stage of tumor growth with destruction of the BBB [52] and peripherally infused CTLs can enter the CNS in patients with malignant glioma [53]. Glioma-derived chemokines such as CCL2, CCL7, or CCL20 can mediate the recruitment of immune cells [54, 55].

## 3. Antitumor Immune Responses of Effector Cells

Effector cells used in ACT for the malignant glioma have developed from lymphokine-activated killer (LAK) cells with nonspecific cytotoxicity to more tumor-specific genetically engineered CTLs over time. The advantages and the disadvantages of the effector cells used in ACT for malignant glioma are summarized in Table 2.

### 3.1. LAK Cells

Autologous LAK cells are a mixture of IL-2 activated T cells and natural killer (NK) cells and are generally obtained by culture of PBMCs in the presence of IL-2. Major therapeutic limitation of these cells against tumors is that their lytic properties are not specifically directed against tumor cells. Autologous tumor cells were usually used as antigen source in ACT using LAK cells for malignant gliomas [56–59].

Although several clinical trials by intratumoral injection of LAK cells combined with IL-2 for the glioblastoma patients have been carried out, most of their therapeutic effects have not shown a significant survival benefit [60–70]. In addition, the use of LAK cells in combination with IL-2 was not superior to the use of IL-2 alone in the phase III trial for other tumors [71]. Moreover, IL-2 related toxicities that emerged in some studies such as brain edema and aseptic meningitis have disturbed widespread use of this strategy for malignant gliomas [63, 65, 70].

### 3.2. NK Cells

In contrast to adaptive immune responses, innate lymphocytes such as NK cells and *γδ* T cells broadly recognize and immediately respond to a certain range of antigens in a MHC-independent fashion [72]. NK cells, CD3^−^CD56^+^ lymphocytes, play potential role in cancer immunosurveillance as innate immune cells. They initially recognize the tumor cells via cellular stress or danger signals. Activated NK cells can directly kill tumor cells without MHC restriction, interact with DCs to facilitate the generation of antigen-specific CTL response by enhancing their antigen uptake and presentation, and induce CD8^+^ T cells to become CTLs by producing cytokines such as IFN-*γ*. Cytokines produced by NK cells can also regulate antitumor antibodies produced by B cells [73–75]. Both allogeneic and autologous IL-2 activated NK cells, furthermore, recognize and kill human glioblastoma cells with stem cell-like properties [76].

Although clinical trials with ACT using LAK cells did not show a significant clinical benefit for malignant gliomas as discussed above, recent advances in NK cell immunobiology and results in animal studies showing favorable antitumor effect in glioma-bearing mice treated with activated NK cells take a growing interest in ACT using activated NK cells again. NK cells can do traffic to the brain directly [77], so both peripheral and intratumoral route of administration are available in the treatment of malignant gliomas. In a rat glioma model, no therapeutic effect was observed in animals treated with intradermally injected paraformaldehyde-fixed tumor vaccine alone, but intratumoral injection of IL-2-activated rat NK cells strongly enhanced antitumor effect of the vaccine [78]. Also, intracranial injection of cytokine-induced killer cells markedly inhibited intracranial xenotransplanted glioma growth in mice [79].

Safe antitumor response was shown in a clinical trial that exclusively used* ex vivo* expanded autologous NK cells to treat recurrent malignant glioma patients [80]. In this study, two (22%) of the nine patients injected focally and intravenously showed partial response. Additionally, prolonged survival of the patients with malignant glioma treated by tumor-loaded DCs vaccine may be associated with NK cell response such as high level of circulating IFN-*γ* and increased NK cell vaccine/baseline (V/B) ratio that was inversely correlated with TGF-*β*2 V/B ratio [81]. These results suggest that a strategy of ACT using* ex vivo* activated NK cells following tumor-loaded vaccine can have a potent antiglioma effect as in animal studies.

Tumor cells, however, have various mechanisms to avoid NK cell recognition including the expression of MHC class I and ligands for inhibitory receptors on NK cells [82, 83]. In order to overcome this resistance of tumor cells to NK-mediated cytotoxicity and enhance tumor recognition of NK cells, gene modification can be utilized. Antitumor activity of NK cells can be enhanced by genetic modification to highly express cytokines, Fc receptors, and/or chimeric antigen receptors (CARs) [84–86]. CAR directly recognizes tumor cell surface antigens and provides specificity of engineered cells regardless of antigen processing or MHC-restricted presentation. Cytokine gene transfer such as IL-2 [87–89], IL-12 [88, 90], IL-15 [91–93], and stem cell factor (SCF) [94] induces NK cell proliferation and survival, and gene transfer of CARs against HERs/neu [95], carcinoembryonic antigen (CEA) [96], and CD33 [97] shows increased specificity [85]* in vitro* and* in vivo* studies. These results suggest ACT using genetically modified NK cells can be a challenge to patients with cancer including malignant gliomas.

NK cell-based immunotherapy has several potential limitations including the immunosuppressive microenvironment of the tumors. Activation of myeloid derived suppressor cells (MDSCs) and Tregs, especially, are known to be major barriers. MDSCs, a heterogeneous population of CD11b^+^, Gr-1^+^ cells of immature myeloid origin, consist of myeloid progenitors and precursors of macrophages, granulocytes, and DCs and have a strong ability to suppress a variety of T cell and NK cell functions [98–100]. MDSCs can also modulate the induction of Tregs [101, 102]. MDSCs increase in malignant glioma-bearing mice [77] and effectively inhibit NK cell-mediated tumor suppression. Circulating number of these tumor suppressor cells also increases in the patients with malignant gliomas [103, 104]. Although there have been no published studies on human glioma-infiltrating MDSCs to date, many preclinical studies to improve antitumor effect by reducing MDSCs in tumor-bearing animal models have been carried out [105, 106].

Tregs are potential inhibitors of NK cell activity in malignant gliomas [107]. Tregs directly inhibit NKG2D-mediated NK cell cytotoxicity, effectively suppressing NK cell-mediated tumor rejection by a TGF-*β* dependent mechanism and independent of IL-10 and depletion of Tregs via NKG2D before NK cell activation markedly enhances NK cell-mediated suppression of tumor growth and metastases in animal studies [108]. Tregs also decrease NK cell cytotoxicity and downregulate the IFN-*γ* secretion of NK cell responding to IL-12 activation in a TGF-*β* dependent manner [109]. Elimination or inhibition of these immunosuppressive cells, therefore, can improve the antitumor effect of ACT using NK cells.

### 3.3. *γδ* T Cells

*γδ* T cells are a subpopulation of T lymphocytes, which express T-cell receptors (TCRs) consisting of one *γ* chain and one *δ* chain. Unlike the conventional *αβ* T cells that recognize only MHC-related antigens, *γδ* T cells can broadly recognize and immediately respond to a range of antigens in a MHC-independent manner.

*γδ* T cells have potent cytotoxic activity against malignant glioma cells [110, 111]. Antiglioma effect of human *γδ* T cells can be increased by the addition of IL-12 [112, 113]. Intracranial infusion of expanded and activated *γδ* T cells can mediate killing of new or established glioblastoma xenografts and reduce tumor progression [114].* Ex vivo* expanded and activated *γδ* T cells from both patients and healthy volunteers can recognize and kill glioblastoma cell lines and primary glioblastoma culture cells, but *γδ* T cell counts and mitogen-stimulated proliferative response of *γδ* T cells are markedly decreased in glioblastoma patients prior to treatment, suggesting that allogeneic therapy could be a reasonable option in adoptive *γδ* T cell immunotherapy [115].

Despite of the theoretical basis of *γδ* T cell-based immunotherapy, there have been no clinical studies designed to assess the immunotherapeutic potential of *γδ* cell therapy against malignant gliomas to date. A recent report that gene modified *γδ* T cells have greater cytotoxicity to temozolomide (TMZ) resistant glioblastoma cell lines in the presence of TMZ than unmodified cells [116] suggests combined TMZ resistant *γδ* T cell immunotherapy and high dose TMZ chemotherapy could be a new therapeutic challenge to the glioblastoma patients.

### 3.4. TILs

Tumor infiltrating lymphocytes (TILs) are effector cells presumably thought to be able to recognize and respond to the specific tumor antigens because they are already present in the tumor. Although antitumor activity of endogenous TILs may not be sufficient to conquer tumor-induced immunosuppressive environment,* ex vivo* expansion of these cells may overcome this immunologic barrier and be a tool of ACT for tumors.* Ex vivo* expanded TILs have the properties to proliferate* in vivo* and display functional activity and trafficking to tumor [117]. Significantly increased antitumor activities of* ex vivo* expanded TILs therapy have been shown in clinical trials for melanoma especially in combination of lymphodepletion with intensive chemoradiation [118, 119].

It is difficult, however, to expand TILs from tumor tissues in most cancers including malignant glioma except melanomas [120]. In a pilot study exclusively performed to date against patients with recurrent malignant gliomas that were treated with intratumoral infusion of* ex vivo* expanded autologous TILs with IL-2, one of six patients showed complete remission, two had partial responses, and three died of tumor progression [56]. The cytotoxic activity of TILs against autologous tumors* in vitro* was variously dependent on the patients and was not correlated with the clinical outcome in this study. These results suggest that clinical benefit from ACT for malignant gliomas using* ex vivo* expanded TILs may be limited.

### 3.5. Antigen-Specific CTLs

Antigen-specific CTLs commonly generated by* ex vivo* antigen stimulation of PBMCs with autologous inactivated tumor cells have potent antitumor immune response compared with T cell response to endogenous stimulation. These CTLs are also able to migrate to antigen-expressed tumor cells following administration and have durable antitumor effect by memory T cells.* Ex vivo* expansion of CTLs for strong priming of T cells with antigens and for rapid increase of effector T cell numbers makes these cells feasible to be used in ACT for cancers.

To date, 4 phase I trials to evaluate CTLs generated from PBMCs [57–59, 121] and 3 phase I and 2 pilot studies examining CTLs obtained by lymphocytes from tumor draining lymph nodes or PBMCs after vaccination with irradiated autologous tumor cells [53, 122–125] against malignant gliomas have been described. Total 9 clinical trials of ACT using antigen-specific CTLs showed 2 complete response (CR), 26 partial response (PR), and 16 stable disease (SD) in 87 patients with malignant gliomas (65 glioblastoma). Data from 49 patients with glioblastoma exclusively in 8 trials except a study that did not describe the results from the distinguished tumor grade [125] demonstrated a result of no CR, 11 PR, and 6 SD. A pilot study for 19 patients with recurrent malignant gliomas (16 glioblastoma) that did not distinguish tumor grade in treatment outcome displayed a favorable result of 1 CR, 7 PR, and 9 SD [125]. More improved median survival of 12 months after tumor recurrence compared with 6 months for controls and a positive correlation between increased survival and delayed-type hypersensitivity response were described in this study [125]. Similarly, a positive correlation between CD4/CD8 composition of infused cells and clinical response was reported [124]. Most other trials, however, did not show survival benefit and a clear association between the concentration of injected T cells and clinical outcome.

### 3.6. CD4^+^ T Cells

CD4^+^ T cells contribute to the immunologic antitumor activity through their ability to mediate tumor cell destruction independent of CD8^+^ T cells as well as help activate CD8^+^ T cells classically [126–128]. Identification of MHC class II-restricted isotopes derived from several TAAs including melanoma differentiation antigens and several cancer-testis antigens becomes feasible to generate antigen-specific CD4^+^ T cells which can be used in ACT [129–131]. Several preclinical studies have described antitumor effect of ACT using CD4^+^ T cell population, and CD4+ T cells have cytolytic activity dependent on class II-restricted recognition of tumors [132–134]. In a recent early-phase dose escalation study of ACT for patient with metastatic melanoma using CD4^+^ T cell clones, the patients experienced partial responses including a case of a complete durable response [128, 135].

## 4. Enhancement of Tumor-Specific T Cell Function

### 4.1. Genetically Modified T Cells

Recently, gene modification of T cells has been developed for enhancing the efficacy of ACT. Gene engineering of T cells by a variety of gene transfer techniques is able to allow T cells to make them more resistant to immune evasion mechanisms of tumor cells or modify the tumor environment to make it less inhibitory to T cell activation and effector function [9] (Table 3). Retroviral or lentiviral vectors are usually used for gene delivery [14, 15].

Two most common approaches can be used for enhancement of T cell specificity: (a) gene modification with TCR variable *α* and *β* chains cloned from high affinity TAA-specific T cells and (b) insertion of chimeric antigen receptors (CARs) that recognize tumors through single-chain variable fragment (scFv) isolated from TAA-specific Abs.

Genes encoding TCRs of T cells isolated from patients showing an excellent response to ACT can be cloned into viral vectors and then be used to alter T cells from other patients with matching HLA restriction elements to be treated [17]. These genes can also be isolated from humanized mice that have been primed to recognize TAAs. Humanized mice that have been cloned human MHC class I or class II molecules can express human MHC molecules and can be immunized with human TAAs of interest. Mouse T cells specific for certain MHC-restricted epitope can then be isolated, and their TCR genes are cloned into viral vectors that can be used to genetically modify T cells from the patient [17, 158].

Some clinical studies for patients with metastatic melanoma using T cells genetically modified with tumor antigen-specific T cell receptors for patient with melanoma have been conducted [16, 17]. In a recent clinical study assessing ACT using a high-avidity TCR recognized MART-1 and gp 100 for patients with metastatic melanoma, cancer regression was seen in 30% and 19% of patients who received the human or mouse TCR, but patients exhibited destruction of normal melanocytes throughout the body including skin, eye, and ear as the result of CTL responses to cognate antigen-containing cells [17]. No clinical study for malignant gliomas, however, has been performed to date. This procedure allows the rapid production of TAA-specific T cells but has a basic limitation that T cells engineered by this procedure can mainly recognize antigens that have processed and presented in MHC-restricted patterns.

An alternative approach to overcome this limitation is the use of CARs, genes encoding monoclonal antibody chains specific for TAAs [18]. T cells modified with CARs can be directed toward any antigen expressed on the cell surface because CARs provide T cell activation regardless of MHC-restricted presentation. CARs are synthetic molecules that consist of an extracellular antigen binding domain that usually contains the heavy and light chain variable regions of a monoclonal antibody, referred to as a single chain Fc (scFv) molecule, joined to transmembrane and cytoplasmic signaling domains derived from CD3-*ζ* chain or Fc receptor *γ* chains (FcR*γ*) and from costimulatory molecules. Engineered T cells activated by both tumor-specific TCR and costimulatory molecules such as CD28, 4-1BB, OX40, and inducible costimulator (ICOS) have enhanced antitumor activity to tumors [23–25, 159, 160].

T cells and expressing CARs for the glioma-specific antigens including IL-13R*α*2, HER2, EGFRvIII, and EphA2 show potent antiglioma activity in preclinical animal studies [19–22, 51]. In a study, T cells from glioblastoma patients could be modified with HER2-specific chimeric antigen receptors to produce effector cells and killed autologous HER2-postive glioblastoma cells including CD133-positive glioblastoma stem cells. These HER2-specific T cells also had a potent antitumor activity against autologous tumors in an orthotopic xenogeneic SCID mouse model [20]. Recently, cytomegalovirus has emerged as a target for the treatment of malignant gliomas. Expression of genes unique to cytomegalovirus (CMV) in malignant gliomas has raised the possibility of CMV-specific T cells as a therapeutic tool [161–164]. Data from a recent clinical study to evaluate antiglioma response of ACT using CMV-specific T cells in combination with TMZ into a patient with recurrent glioblastoma showing a long-term disease free survival [164] suggest CMV can be a challenging target of ACT for malignant gliomas and provide an important clue for further evaluation of combined ACT and TMZ chemotherapy.

Although clinical experience of ACT using T cells expressing TAA-specific CARs is limited, therapeutic limitations of these cells have emerged. In a clinical study targeting three glioblastoma patients treated by intracranial adoptive transfer of autologous IL-13R*α*2-specific CTL clones, safe antiglioma responses against antigen positive CD133^+^glioma stem cells as well as antigen positive glioma cells were documented, but IL-13R*α*2 antigen was not expressed in the eventually recurred tumor [165]. Immune escape like these antigen loss variants also can be presented in peptide vaccination targeting EGFRvIII in patients with glioblastoma [166], so antigen loss variants may be a major mechanism responsible for tumor progression.

In addition, there are safety concerns with regard to HER2-targeted T cell therapy. A patient administered T cells with a CAR recognizing ERBB2 died of respiratory distress probably due to cytokine storm by massive release from ERBB2 expressing T cells localized to the lung with recognition of low levels of ERBB2 on lung epithelial cells [167].

Genetic engineering can increase effector function of T cells by modification of tumor environment as well as enhanced T cell specificity to malignant gliomas. Other strategies for cancers to increase T cell effector function through genetic modification are described (Table 3).

### 4.2. Prolongation of T Cell Survival

Identification of T cell populations that can reproducibly survive* in vivo* for increased antitumor effect in ACT is also important. CD8^+^ T cells have been described as naive cells and four antigen-experienced subtypes according to the differentiation status: T memory stem cell (T_SCM_), central memory (T_CM_), effector memory (T_EM_), and differentiated effector T cells [168]. T cell differentiation is inversely correlated with antitumor effect in ACT for cancer [158, 169]. Preclinical studies in human T cells suggest that arrested differentiation via reducing IL-2 concentration in culture condition [170–172] and inhibitors of the WNT signaling pathway [173, 174] can lead to enrichment of less differentiated memory T cells with high replicative potential.

Recently isolated T_SCM_ cells in mouse model, the least differentiated memory subset, have a preferential intrinsic capacity for long-term* in vivo* persistence and for self-renewal, and a multipotent ability to derive T_CM_, T_EM_, and effector T cells in response to antigen reexposure [168, 175]. T_SCM_ cells have been shown to be more effective than T_CM_ cells which were more effective than T_EM_ cells in terms of ACT against tumors in various preclinical studies [135, 158, 169, 176]. T_SCM_ cells consistently express a surface marker typically found on naive T cells and also express stem cell antigen-1 (Sca-1), B cell lymphoma 2 (Bcl-2), the *β* chain of the IL-2 (IL-2R*β*), and the chemokine (C-X-C motif) receptor CXCR3 [168, 177]. The identification and* ex vivo* expansion to minimize corruption of a similar human stem cell-like memory T cells may be important in the development of ACT, and these cells may play a greater role in human future ACT strategies for patients with cancer.

## 5. Modification of the Host Environment

### 5.1. Lymphodepletion

Lymphoid cells have an independent homeostatic regulation of resting and memory cell compartments, so a rapid proliferation of remaining or infused lymphocytes happens to recover normal lymphocyte numbers after periods of lymphopenia [178, 179]. During homeostasis-induced T cell proliferation, naive T cells stably acquire the cell surface markers and functional properties of memory T cells capable of rapid and intense response to antigen, and these homeostasis-stimulated memory CD8^+^ T cells respond to lower doses of antigen than naive cells [180]. Considering that this recovery is mediated by MHC dependent recognition, that memory CD8^+^ T cells respond in the reduced activation threshold of tumor-specific cells, and that proliferated T cells have effector functions, administration of tumor specific antigens in the form of a vaccine or* ex vivo* expanded adoptive T cell transfer during this recovery period can induce disproportionate enhancement of effector cell populations that have autoimmune responses against tumor-associated self-antigens, leading to increased antitumor effect of ACT [180–184].

The induction of immunodepleting condition in patients before T cell-based immunotherapy can be achieved by use of total body irradiation (TBI) or nonmyeloablative chemotherapy. Data in clinical trials using these approaches have been shown to enhance the efficacy of ACT [118, 119, 185–188] such as melanocyte-directed autoimmunity noted in some patients with metastatic melanoma treated by these approaches [118, 189].

Another therapeutic advantage of lymphodeletion prior to immunotherapy is the elimination of major immunosuppressive cellular elements within the tumor microenvironment such as MDSCs and Tregs. As described above, MDSCs are found in most patients with advanced cancers [103, 190–192], so elimination or blockade of the immunosuppressive functions of MDSCs can provoke an enhanced antitumor effect of immunotherapeutic strategies for tumors [193, 194]. MDSCs can also modulate the induction of Tregs [101, 102]. Tregs that play a two-directional role in controlling autoimmunity and T cell homeostasis can selectively suppress spontaneous lymphopenia-induced naive T cell proliferation [195] and actually enhance immune function by optimization of the conventional T cell diversity [196]. Tregs are increased after total body irradiation and inhibit the induction of effector T cells during recovery period from lymphopenia, whereas depletion of Tregs strongly inhibits tumor progression in animal study [197]. In a recent clinical pilot study, anti-IL-2R*α* MAb daclizumab treatment combined with EGFRvIII-targeted peptide vaccination could deplete Tregs safely and significantly in patients with glioblastoma treated with lymphodepleting TMZ correlating with enhanced antitumor immunity [198].

Additionally, ACT can be enhanced by the increased depletion of endogenous cells that compete for homeostatic cytokines such as IL-7 and IL-15 [119], by the promotion of the expansion and function of adoptively transferred antitumor CD8 T cells through hematopoietic stem cells [199], and by the increased functionality of adoptively transferred T cells mediated by TBI-evoked microbial translocation [200].

### 5.2. Inhibition of Immunosuppressive Environment

Elimination or blockade of immunosuppressive molecules of human cancers can enhance the antitumor efficacy of ACT. The challengeable targets for the treatment of malignant gliomas can be TGF-*β*, Tregs, and signal transducer and activator of transcription 3 (STAT3).

TGF-*β* is a potent immunodepressant and blocking of TGF-*β* effects on T cells can improve antitumor efficacy of T cells after ACT for malignancies [153, 154, 201]. Administration of TGF-*β* receptor I kinase inhibitor increases tumor infiltration by NK, T cells and macrophage and increases survival in glioma-bearing mice [202, 203]. The most clinically advanced strategy to elicit TGF-*β* in gliomas is the use of intratumorally administered TGF-*β*2 antisense oligonucleotides using convection-enhanced delivery [204]. Phase II study that evaluated the efficacy and safety of trabedersen (TGF-*β*2 antisense oligonucleotides) administered intratumorally by convection-enhanced delivery compared with standard chemotherapy in patients with recurrent malignant gliomas showed a superior safety and a trend for superiority in 2-year survival rate of patients with anaplastic astrocytoma compared to chemotherapy [205]. However, further clinical study discontinued during the phase III trial unfortunately. TGF-*β* also influences the development, maintenance, and induction of Tregs, while disruption of TGF-*β* signaling prevents the generation of Tregs [206, 207].

Tregs have an important role in maintaining self-tolerance and in the prevention of autoimmunity physiologically, and increased Tregs fractions with CD4^+^ T cell defects inducing decreased T cell responses are seen in patients with gliomas [208]. Characteristics of Tregs in both mice and humans are the high expression of surface markers CD25 (IL-2R-*α*-chain), constitutive expression of cytotoxic T-lymphocyte antigen 4 (CTLA-4), overexpression of glucocorticoid-induced tumor necrosis factor receptor-related protein (GITR), and the expression of the transcriptional regulator Foxp3 [209, 210]. These molecules can be therapeutic targets for depleting Tregs to improve ACT for gliomas.

Strategies such as anti-CD25 antibody and CD25-specific immunotoxin [211] employed to reduce Treg function target the constitutively expressed cell surface marker, CD25. IL-2R*α* (CD25) blocking with anti-IL-2*α* (anti-CD25 antibody) daclizumab combining glioma antigen (CMV or EGFRvIII) specific vaccination during lymphopenia selectively depletes Tregs in mice and humans [198, 212].

Another possible approach to reduce Tregs in glioma is via CTLA-4 blockade. CTLA-4 is a transmembrane protein that binds to ligands B7-1 and B7-2 on APCs and is constitutively expressed on Tregs, acting as a potent negative regulator of T cell activation. Anti-CTLA-4 antibodies have shown potential therapeutics for gliomas [213], and combining sequential immunotherapy with GM-CSF expressing irradiated glioma cell vaccine synergistically prolongs survival in mice-bearing gliomas [214].

STAT3 is generally overexpressed in cancers including malignant gliomas and plays an important role in negative regulation of antitumor immunity. STAT3 regulates the expression of TGF-*β* and IL-10, cytokine related to the presence of Tregs in tumors, so STAT3 can be a target for depleting Tregs. Inhibition of STAT3 promotes the activity of NK and T cells on cancer cells [215, 216]. STAT3 inhibition was shown to reverse the immunosuppressive environment in malignant gliomas [217] and to promote the efficacy of ACT in a murine glioma model [216]. Furthermore, adoptive transfer of T cells that transfected miRNAs, gene transcripts modulating STAT3 signaling, exerts potent antiglioma therapeutic effects in genetically engineered murine glioblastoma models and enhances effector responses in the local tumor microenvironment [218]. Additionally, a low dose metronomic TMZ therapy can induce Treg depletion [219] and inhibit trafficking of Tregs into the glioma microenvironment [220].

IDO is an intracellular enzyme that catalyzes oxidative catabolism of tryptophan [221, 222]. T cell proliferation is arrested when exposed to tryptophan shortage evoked by IDO, and most human tumors including gliomas evade cellular immune response through the constitutively expressed IDO [223]. Consequently, IDO expressing tumor cells are able to inhibit tumor specific T cell response [224]. Expression of IDO in APCs also allows macrophages and DCs to inhibit T cell proliferation [225] and expand potent autologous Tregs [226]. Inhibition of IDO can improve T cell therapy for cancers [227–229]. In addition, molecular targeted therapy with imatinib can potentiate antitumor cell responses in gastrointestinal tumor through the inhibition of IDO [230].

Recently, IDO emerged as therapeutic target for the treatment of gliomas [231]. IDO expression in glioma is associated with malignant progression [232] and a significant decrease of overall survival in patients [233]. IDO expression in brain tumors also increases the recruitment of Tregs in mouse model [233, 234].

## 6. Combining T Cell Therapy

Combining immunotherapy with cytotoxic chemotherapy or targeted therapy can promote the therapeutic potential for the treatment of cancers in comparison with the use of either treatment alone because abundant antigens can be released from the dying tumor cells and increased effector cell capacity to recognize and kill tumor cells can be induced by cytotoxic chemotherapeutic agents [235, 236]. This antigen processing can lead to the priming of adoptively transferred tumor-specific T cells as well as the activation of endogenous tumor-specific T cells. Chemotherapy can enhance tumor cell susceptibility to CTL-mediated cytotoxicity during cancer immunotherapy, increasing the efficacy of tumor-specific T cell activation in mice with advanced cancer [237, 238]. Furthermore, chemotherapy (dacarbazine, temozolomide, and cisplatin) induces intratumoral expression of T cell attracting chemokines [239]. Combined TMZ chemotherapy and immunotherapy with DC-based vaccines can lead to the enhancement of antitumor immunity through increased tumor-specific immune responses via the cross-priming of apoptotic tumor cell death as well as suppression of Tregs in glioma bearing mice [26] and showed to be beneficial for survival in a phase II trial in patients with newly diagnosed glioblastoma [27].

Oncogene addiction is a phenomenon in which the survival of cancer cells depends on an activated oncogene or inactivation of tumor suppressor gene and is an ideal potential target for molecular targeted therapy in human cancers [240, 241]. Tumor cell death after oncogene addiction may provide antigenic stimulation of T cells, and oncogene addiction may also reduce the production of immunosuppressive molecules by tumor cells, promising increased antitumor efficacy of combining ACT with molecular targeted therapy for cancers including gliomas [242]. Actually, BRAF inhibition can induce the enhanced T cell recognition and subsequent T cell response on melanoma cells [243], and BRAF inhibitor vemurafenib improves the antitumor activity of ACT for advanced melanoma in mice [244].

*In vivo* expansion of T cells by vaccination has limitation due to the immunosuppressive environment of the tumor, and clinical trials using vaccine alone do not have significant antitumor effect [245]. Combining T cell therapy and vaccination can also be an alternative approach to facilitate expansion and maintenance of T cells that survived in poor immunogenic tumor environment.

## 7. Future Directions

T cells used in ACT for malignant gliomas have been developed and will be more advanced to overcome immune evasion mechanisms and to survive in immunosuppressive environment employed by the tumor.

Future efforts will need to focus on identification of patient-specific tumor antigens through highly personalized approach, development of efficient lymphodepleting regimens prior to T cell transfer, and effective combination with other therapeutic modalities such as molecular agents targeting personalized oncogene addiction and potent host immune modulators.

## Figures and Tables

**Table 1:** Glioma-associated antigens.

Classification	Antigens [references]
Mutated antigens	EGFRvIII [35]
Cancer-testis antigens	MAGE [36], GAGE [37], and SOX6 [38]
Tissue-specific antigens	Gp100 [39], TRP-2 [40]
Others	IL-13R*α*2 [41], EphA2 [42], EphB6 [43], HER-2[39], AIM-2 [44], SOX11 [45], surviving [46], telomerase [47], Mart-1 [48], and KIF3C [49]

**Table 2:** Comparison of the effector cells used in adoptive T cell therapy for malignant glioma.

Effector cells	Advantages	Disadvantages
Lymphokine-activated killer (LAK) cells	MHC-independent cytotoxicity Easy preparation of cells	Nonspecific killing IL-2 related toxicities

Natural killer (NK) cells	MHC-independent cytotoxicity Immediate response Can be modified to target tumor antigens genetically	Nonspecific killing

*γδ* T cells	MHC-independent cytotoxicity Immediate response	Nonspecific killing

Tumor infiltrating lymphocytes (TILs)	Presumably tumor-specific killing	Need T cells from tumor tissue Technical difficulty to expand *ex vivo *

CD4^+^ cytotoxic T lymphocytes	Tumor-specific killing	MHC class II-dependent cytotoxicity

CD8^+^ cytotoxic T lymphocytes	Tumor-specific killing Can be modified to target tumor antigens genetically	MHC class I-dependent cytotoxicity

Genetically modified cytotoxic T lymphocytes	MHC-independent cytotoxicity Rapid and elaborate tumor-specific killing	Induction of antigen loss variants at tumor recurrence Possible overreactivity on same target antigens expressed in normal tissue

**Table 3:** Genetic modification of T cells to improve the efficacy of ACT for cancers.

	References
Enhanced specificity	
Expression of *αβ* TCR	[16, 17, 136]
Expression of CARs	[18–22]
Coexpression of costimulatory molecules	[23–25, 137]
Increased survival and proliferation	
Expression of proliferative cytokines	[138–141]
Expression of antiapoptotic genes	[142–144]
Ectopic expression of gene for telomere elongation (hTERT)	[145–148]
Enhanced trafficking	
Expression of chemokine receptors	[149–152]
Enhanced trafficking	
Expression of negative TGF-*β* receptor	[153–156]
Downregulation of Fas	[157]
Integration with conventional therapy	
Expression of chemoresistant genes	[116]
